# Human Herpesvirus 6B U26 Inhibits the Activation of the RLR/MAVS Signaling Pathway

**DOI:** 10.1128/mBio.03505-20

**Published:** 2021-02-16

**Authors:** Xuefeng Jiang, Tian Tang, Jinfeng Guo, Yuhang Wang, Peipei Li, Xiangjun Chen, Lily Wang, Yiqun Wen, Junli Jia, Garbarino Emanuela, Benshun Hu, Shuhua Chen, Kun Yao, Linyun Li, Huaming Tang

**Affiliations:** a Department of Immunology, Nanjing Medical University, Nanjing, People’s Republic of China; b Department of Women’s Hospital of Nanjing Medical University, Nanjing Medical University, Nanjing, People’s Republic of China; c Department of Critical Care Medicine, Changzhou Cancer Hospital Affiliated to Soochow University, Changzhou, People’s Republic of China; d Department of Medical Genetics, Nanjing Medical University, Nanjing, People’s Republic of China; e Key Laboratory of Antibody Technique of Ministry of Health, Nanjing Medical University, Nanjing, People’s Republic of China; f The Laboratory Center for Basic Medical Sciences, Nanjing Medical University, Nanjing, People’s Republic of China; Virginia Polytechnic Institute and State University

**Keywords:** HHV-6B, U26, RLR/MAVS signaling pathway

## Abstract

U26 is one of the roseolovirus unique genes with unknown function. Human herpesvirus 6B (HHV-6B) pU26 is predicted to be an 8-transmembrane protein containing a mitochondrion location signal. Here, we analyzed U26 function during HHV-6B infection and find that (i) HHV-6B U26 is expressed at a very early stage during HHV-6B infection, and knockdown of it results in a significant decrease of HHV-6B progeny virus production; (ii) U26 inhibits the activation of the retinoic acid-inducible gene I (RIG-I)-like receptor (RLR)/mitochondrial antiviral signaling protein (MAVS) signaling pathway, an important anti-HHV-6B infection innate immune response, by targeting MAVS protein for degradation; and (iii) a portion of U26 locates to the mitochondria, which could affect the mitochondrial membrane potential and finally leads to MAVS degradation. These findings indicate that HHV-6B U26 is a novel antagonistic viral factor against host innate antiviral immunity.

## INTRODUCTION

Human herpesvirus 6 (HHV-6), together with HHV-7, belongs to the *Roseolovirus* genus of the betaherpesvirus subfamily (1, 2). HHV-6 is a human pathogen with increasingly important clinical significance (3, 4). In 1986, HHV-6 was first isolated from peripheral blood mononuclear cells of AIDS-associated non-Hodgkin lymphoma patients (5). In later studies, it was found that two distinct variants exist, named HHV-6A and HHV-6B (6–8). Now, they are reclassified into two virus species due to genomic differences, distinct epidemiologies, disease associations, biological and immunological properties, and cell tropism (9). These two viruses share ∼90% genomic identity (10). While the diseases caused by HHV-6A are unknown, HHV-6B primary infection occurs mainly in young children and causes exanthema subitum, an acute febrile illness (11). When the host is subjected to immunocompromised or immunosuppressed conditions (12), the latent virus will be activated again, or the exogenous virus infects the host again, causing secondary infection leading to serious diseases, especially in bone marrow suppression, organ transplantation, and AIDS and other immunosuppressed patients (13). Recently, it was reported that HHV-6 could be one of the pathogenic agents of Alzheimer’s disease (14).

The innate immune response is the first line of host defense against incoming microbial infection (15, 16). After virus infection, viral nucleic acid can be detected by various pattern recognition receptors (PRRs) (17). Following recognition, the antiviral immune response will be activated, which results in the production of type I interferons (IFNs) and other cytokines to achieve the inhibition and/or elimination of the virus infection. Generally, among PRRs, some Toll-like receptors (TLRs) and/or retinoic acid-inducible gene I (RIG-I)-like receptors (RLRs) recognize viral RNAs during many virus infections (18). RLRs, which include RIG-I, melanoma differentiation-associated gene 5 (MDA5), and the Laboratory of Genetics and Physiology 2 gene (LGP2), recruit and activate mitochondrial antiviral signaling protein (MAVS) localized at the mitochondrial outer membrane. MAVS plays an important role in antiviral immunity and autoimmunity (19, 20). There are two pathways to activate the downstream reaction of MAVS. The first one involves MAVS recruitment of downstream TANK-binding kinase 1 (TBK1), followed by TBK1 phosphorylation of interferon regulatory factor 3 (IRF3), which eventually stimulates the production of proinflammatory cytokines such as IFN-β and interleukin-6 (IL-6). The second pathway involves MAVS liberation of NF-κB from the IκB kinase α (IKKα)/IKKβ/NEMO complex. NF-κB enters the nucleus and turns on the expression of a cluster of genes, which is essential for immune and inflammatory responses (21).

During viral infections, different antiviral DNA and RNA responses via PRRs are activated (17). In the case of infection of host cells by many DNA viruses, DNA sensors such as TLR9 and cyclic GMP-AMP synthase (cGAS) are activated. Subsequently, signals are transduced via downstream adaptors such as myeloid differentiation factor 88 (MYD88) and stimulator of interferon genes (STING), which is an endoplasmic reticulum (ER)-resident adaptor (22, 23). Most RNA viruses are recognized by a specific set of RNA sensors. RLRs are such sensors, and they play a vital role during RNA virus infection (24–26). Interestingly, during several DNA virus infections, an antiviral role of the RLR signaling pathway has also been reported.

Kaposi’s sarcoma-associated herpesvirus (KSHV) is recognized by both RIG-I and MDA5 via sensing of host RNAs during virus infection (27). During human cytomegalovirus (HCMV) infection, the RIG-I/MAVS signaling pathway is important for antiviral immune responses (28). It has been reported that HHV-6 infection could modulate CD4^+^ T cells’ intracellular TLR9 expression and TLR9-mediated signaling for the development of an effective immune response (29). It is still unknown whether RNA sensors function during HHV-6 infection.

Multiple immune escape strategies are employed by individual viruses to evade detection by the host immune system. HCMV US9 locates to the mitochondria and inhibits the activation of IRF3 by inducing MAVS leakage from the mitochondria (28). The herpes simplex virus 1 (HSV-1) tegument protein UL46 interacts with both STING and TBK1 via separate domains and possibly disrupts their functions to block this DNA-sensing pathway (30). HSV-1 US11 interacts with endogenous RIG-I and MDA5 through its C terminus and inhibits the downstream activation of the RLR signaling pathway (31). An HHV-6 gene cluster, located in U20s of the HHV-6 genome, encodes viral proteins responsible for immune evasion by the virus. U20 is necessary for HHV-6-induced inhibition of tumor necrosis factor (TNF) receptor-dependent signaling and apoptosis (32). An HHV-6 U21 homolog is present in HHV-7, and both of them could divert major histocompatibility complex (MHC) class I molecules to a lysosomal compartment, which contributes to recognition escape by cytotoxic T lymphocytes (CTLs) (33, 34). U24 inhibits CD3 recycling to the cell surface and mediates T cell receptor (TCR) at early endosomes, which prevents the activation of T cells (35). It is still unknown whether there is a viral anti-nucleic acid sensing factor responsible for HHV-6 immune evasion.

Here, we have analyzed HHV-6B U26 function in detail and found that a portion of the HHV-6B U26 protein (pU26) was expressed in mitochondria and that pU26 inhibited the phosphorylation of IRF3 and the subsequent production of type I interferon by targeting MAVS protein to the proteasome degradation pathway. Knockdown of U26 expression resulted in the activation of the RLR/MAVS signaling pathway and decreased the production of progeny HHV-6B. In conclusion, we have identified a novel anti-HHV-6 infection pathway in host cells and also revealed that HHV-6B pU26 counteracts the antiviral RLR/MAVS signaling pathway immune response.

## RESULTS

### Identification of the full-length HHV-6B U26 transcript.

**FIG 1:**
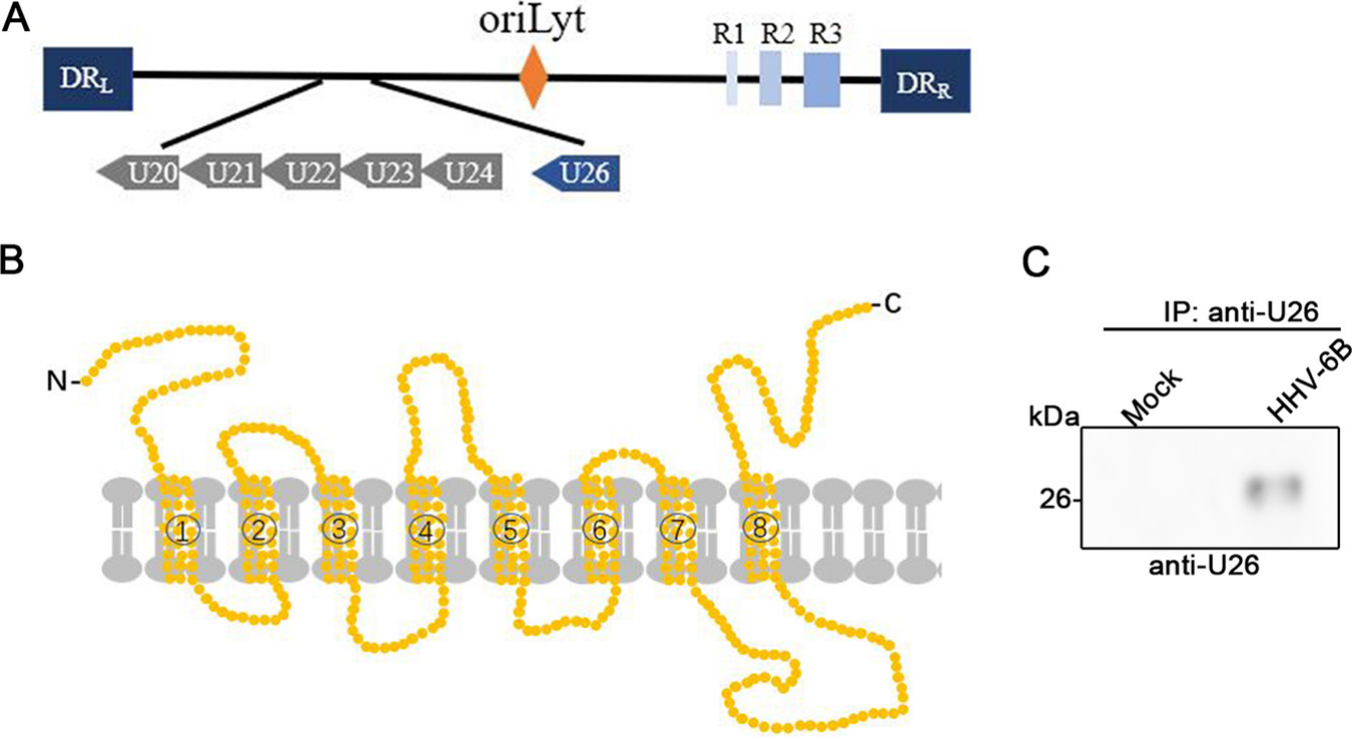
Schematic representation of HHV-6B U26. (A) Schematic representation of the full-length HHV-6B U26 ORF in the HHV-6 genome. The HHV-6B genome comprises three major internal repeat elements (R1 to R3), the origin of replication (oriLyt), and the DR (direct repeat) termini (DR_L_ and DR_R_). (B) Schematic of pU26, which was predicted to have 8 transmembrane domains by the SOSUI website (http://harrier.nagahama-i-bio.ac.jp/sosui/). (C) Mock- and HHV-6B-infected cells were lysed and subjected to immunoprecipitation (IP) with anti-U26 antibody followed by Western blotting with the same antibody.

In order to explore the role of U26 during HHV-6B infection, we first cloned the U26 open reading frame (ORF) according to the sequence deposited in the NCBI database (GeneID 1497028) from a cDNA library constructed by using HHV-6B-infected cells. To confirm the 3′ and 5′ ends of the U26 mRNA, we used 3′ rapid amplification of cDNA ends (RACE) and 5′-RACE analyses, respectively, according to the manufacturer’s protocol. The results confirmed that the full-length U26 ORF was identical to that in the database. The U26 protein was predicted to have 8 transmembrane domains according to the SOSUI website (http://harrier.nagahama-i-bio.ac.jp/sosui/) (Fig. 1A and B). We also analyzed the expression of U26 protein in HHV-6B-infected cells. An ∼25-kDa protein was detected (Fig. 1C).

### U26 is required for HHV-6B propagation.

**FIG 2:**
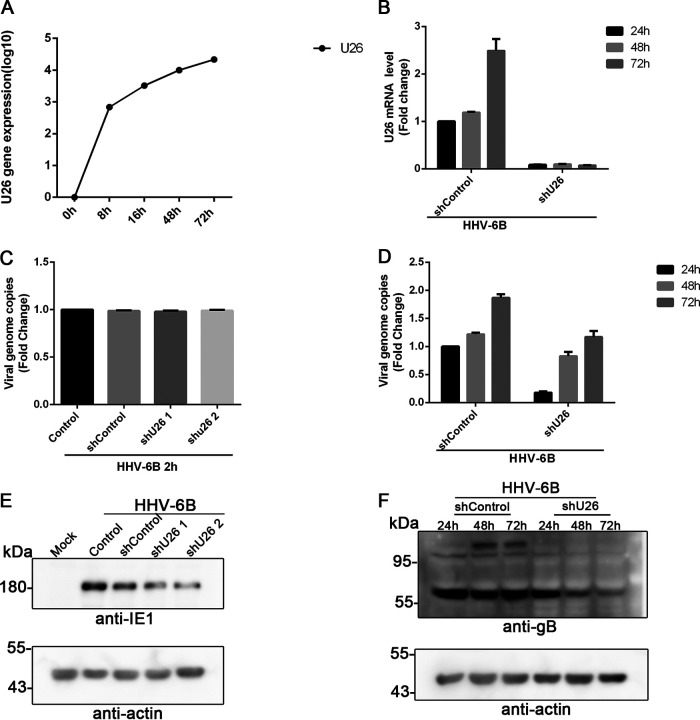
U26 is required for HHV-6B propagation. (A) Growth kinetics of HHV-6B U26. MT4 cells were infected with HHV-6B and harvested at 0 h, 8 h, 16 h, 48 h, and 72 h postinfection. The viral genome copy number in each sample was quantitated by real-time PCR. (B) U26 knockdown efficacy was confirmed by RT-qPCR. MT4 cells were transduced U26 shRNA-expressing lentivirus (shU26) or control lentivirus (shControl), infected with HHV-6B, and harvested at 24 h, 48 h, and 72 h postinfection. Total RNA was extracted from each sample, and U26 expression was measured by RT-qPCR. (C) U26 knockdown had minor effects on the HHV-6B entry process. U26 knockdown cells and control cells were infected with HHV-6B for 2 h at 37°C and then washed with 0.25% trypsin-EDTA. Total DNA was extracted from the infected cells, and real-time PCR was used to measure incoming virus genomes. (D) U26 affected HHV-6B propagation. HHV-6B infected U26 knockdown cells and control cells for 24 h, 48 h, and 72 h. Real-time PCR was used to detect HHV-6 genome copies in culture medium. (E and F) U26 affected the expression of viral proteins. HHV-6B infected MT4 cells, which were transduced with U26 shRNA-expressing lentivirus or control lentivirus and harvested at 24 h, 48 h, and 72h postinfection. Cell lysates were resolved by SDS-PAGE, followed by Western blotting with anti-IE1 (E) and anti-gB (F) antibodies.

The expression kinetics of U26 during HHV-6B infection were analyzed by reverse transcription-quantitative PCR (RT-qPCR). U26 mRNA was detected at the early stage of HHV-6B infection (even at 8 h postinfection) and was dramatically increased following virus propagation (Fig. 2A).

As there is still no effective gene knockout system for HHV-6B gene function analysis, we transduced HHV-6B-permissive cells (MT4 cells) with U26 short hairpin RNA (shRNA)-expressing lentivirus or its control lentivirus, followed by HHV-6B infection. RT-qPCR analyses confirmed the efficacy of U26 knockdown because U26 expression was significantly downregulated compared with the control shRNA-expressing cells (Fig. 2B). We also analyzed whether virus entry was affected by U26 knockdown by measuring incoming virus genomes in the infected cells at 2 h postinfection. The results showed similar entry efficiencies in U26 knockdown cells and control cells (Fig. 2C). Furthermore, we investigated virus propagation by measuring HHV-6B genome copies in the cell culture medium. We found that a retarded viral genome increases in U26 shRNA-expressing cells compared with that in control cells (Fig. 2D). We also detected viral protein expression in HHV-6B-infected cells and found that the expression of the immediate early 1 (IE1) protein and glycoprotein gB was reduced in U26 shRNA-expressing cells (Fig. 2E and F). These results indicated that U26 plays an important role in HHV-6B infection.

### A fraction of U26 is located in mitochondria.

**FIG 3:**
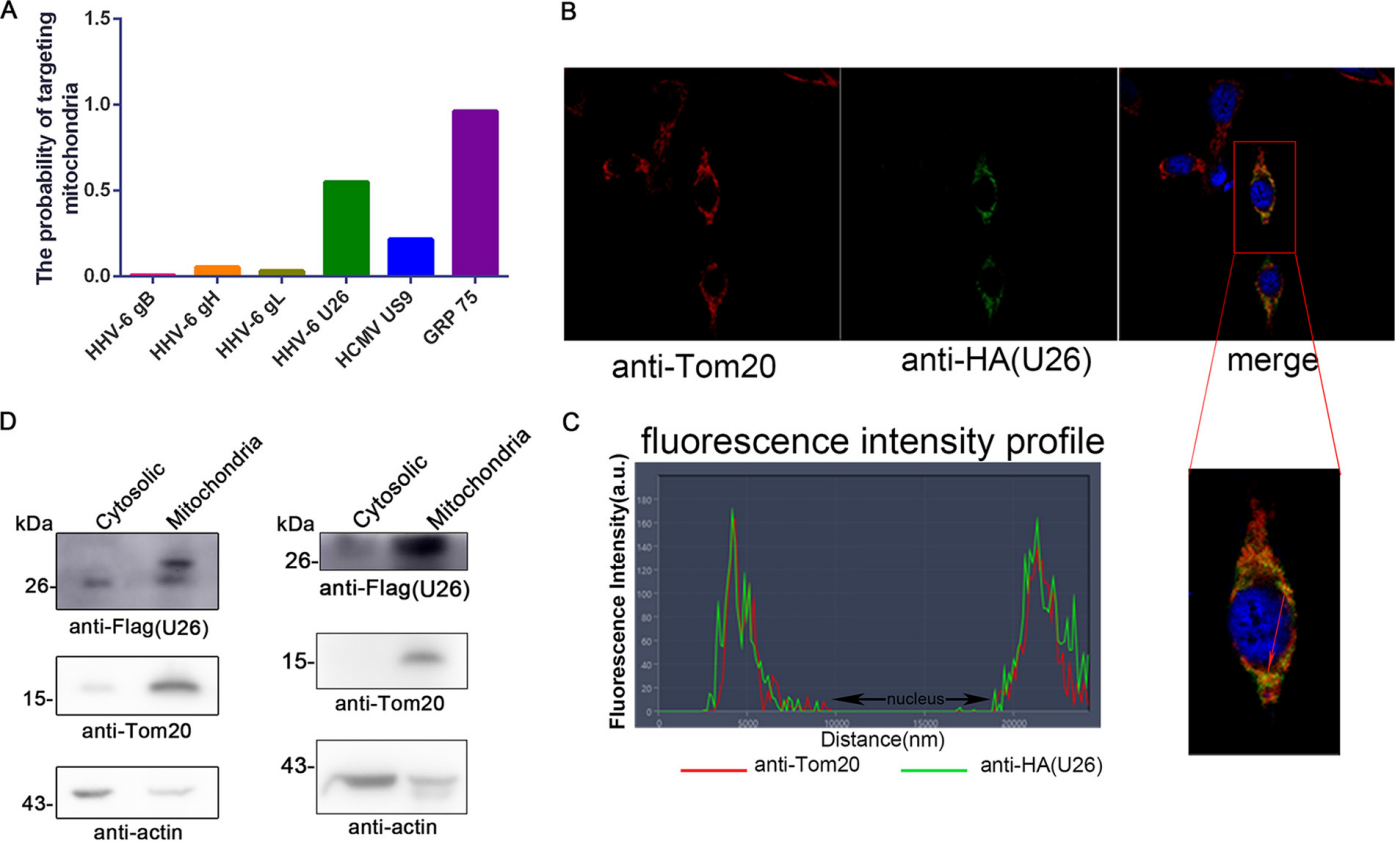
A fraction of U26 is located in mitochondria. (A) Chart showing the possibility of different proteins localizing to mitochondria, as predicted by a mitochondrial prediction website (https://ihg.gsf.de/ihg/mitoprot.html). (B) Confocal microscopy of U26 subcellular localization. HeLa cells expressing HA-U26 were stained with a mitochondrial marker (anti-Tom20) followed by Alexa Fluor 555 goat anti-rabbit secondary Ab (red). These cells were also stained with anti-HA followed by fluorescein isothiocyanate (FITC) 488 goat anti-mouse secondary Ab (green). The nuclei were stained with DAPI (blue). (C) The degree of colocation between U26 and mitochondria was demonstrated by fluorescence intensity profile analysis across the arrowed line (red) in panel B. a.u., arbitrary units. (D) Cytosolic/mitochondrial fractions isolated from U26-Flag-expressing HEK293T cells (left) and HHV-6B-infected cells (right) were immunoblotted with individual antibodies. Anti-Tom20 and antiactin were used as the mitochondrial and cytosolic markers, respectively.

In order to study the U26 function, we first analyzed the predicted intracellular localization of HHV-6B U26 using tools available at https://ihg.gsf.de/ihg/mitoprot.html. We found that U26 had a probability score of 55% for its location in mitochondria, which was much higher than those of the other control proteins, HHV-6 gH, gL, and gB, known as viral envelope glycoproteins, and higher than that of the HCMV US9 protein reported to be located in mitochondria (28). GRP75, a mitochondrial marker, had the highest score, 96% (Fig. 3A). To confirm the prediction result, we performed confocal microscopy analysis and found that a portion of U26 was indeed enriched in mitochondria and overlapped a mitochondrial marker, Tom20, although much of U26 was expressed in the cytosol (Fig. 3B and C). To further confirm its subcellular localization, we isolated cytosolic and mitochondrial fractions from U26-expressing HEK293T cells, analyzed each fraction with antibodies (Abs) for actin (cytosol) and Tom20 (mitochondria) by Western blotting, and found that pU26 was mainly detected in the mitochondrial fraction. Interestingly, two forms of U26 were expressed in U26-transfected cells (Fig. 3D, left) but not in HHV-6B-infected cells (Fig. 1C and Fig. 3D, right), and the relatively large size of U26 was almost completely expressed in the mitochondrial fraction (Fig. 3D, left). How these two sizes of U26 formed still needs to be elucidated. These results indicated that pU26 is expressed and may function in mitochondria.

### U26 downregulates MAVS expression.

**FIG 4:**
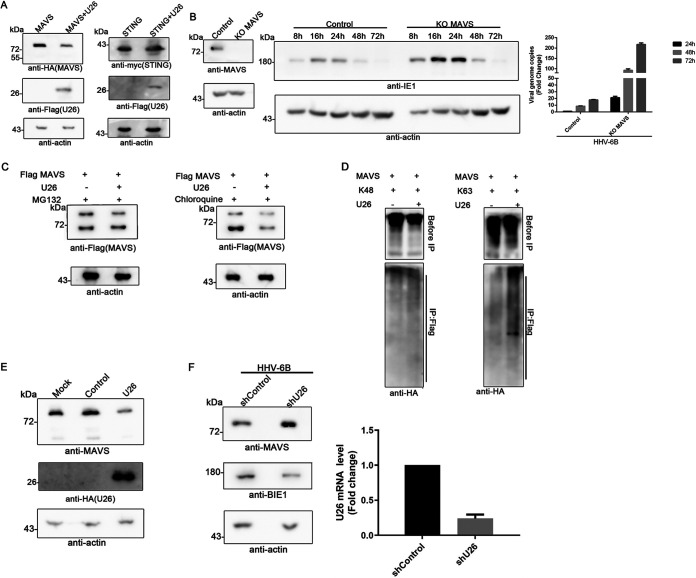
U26 downregulates MAVS expression. (A) U26 decreased the expression of exogenous MAVS without affecting STING expression. HEK293T cells were transfected with the plasmid for either HA-tagged MAVS or Myc-tagged STING expression alone or together with Flag-tagged U26. Cell lysates were resolved by SDS-PAGE, followed by immunoprecipitation with anti-Flag, anti-HA, and anti-Myc. (B) MAVS affected virus propagation. (Left) MT4 cells were lentivirally transduced with MAVS gRNA or control gRNA and selected with puromycin (0.5 μg/ml). MAVS knockout (KO) efficacy was confirmed by Western blotting. (Middle) MAVS knockout cells and control cells were infected with HHV-6B and harvested at 8 h, 16 h, 24 h, 48 h, and 72 h postinfection. The cell lysates were detected by Western blotting with anti-IE1 and antiactin. (Right) Real-time PCR was used to detect HHV-6 genome copies in infected cells and culture medium. (C) U26-mediated MAVS reduction was dependent on the proteasome pathway rather than lysosome pathways. HEK293T cells expressing Flag-tagged MAVS and HA-tagged U26 were treated with MG132 (left) or chloroquine (right) for 4 h. (D) U26 accelerated the ubiquitination of MAVS. HEK293T cells were transfected with plasmids encoding Flag-tagged MAVS together with HA-tagged K48 ubiquitin or HA-tagged K63 ubiquitin. The cells were prepared for immunoprecipitation assays with anti-Flag antibody followed by immunoblot analysis with the indicated antibodies. (E) Detection of endogenous MAVS protein. Cell lysates of U26-expressing HEK293T or control cells were immunoblotted with anti-MAVS. (F, left) Control shRNA- or U26 shRNA-expressing MT4 cells were infected with HHV-6B for 24 h, and endogenous MAVS was detected with anti-MAVS by immunoblot analysis. (Right) U26 knockdown efficacy was confirmed by RT-qPCR.

In previous studies, it has been reported that many viral proteins located in mitochondria affect MAVS expression, which in turn inhibits the RLR/MAVS signaling pathway and ultimately regulates host innate immunity (e.g., HCMV US9 and influenza A virus protein M2) (25, 28). We examined whether U26 functions in a similar way during HHV-6B infection by transfecting HEK293T cells with the plasmid for either hemagglutinin (HA)-tagged MAVS or Myc-tagged STING expression alone or together with U26. We assessed MAVS and STING expression by immunoblot assays and found that U26 dramatically reduced MAVS expression but not STING expression (Fig. 4A). To explore whether MAVS was an antiviral factor during HHV-6 infection, we knocked out MAVS in MT4 cells by using CRISPR-Cas9 technology and analyzed HHV-6 infection in these cells. MAVS knockout was confirmed by Western blotting (Fig. 4B, left). We infected MAVS knockout cells and control cells with HHV-6B and measured virus propagation by detecting viral genome replication and viral protein expression. We found that both virus genome replication and viral protein synthesis were significantly upregulated when MAVS was knocked out compared to those in control cells (Fig. 4B, right). We also measured incoming virus genomes in the infected cells at 2 h postinfection to analyze whether virus entry was affected by MAVS knockout. Our results showed that knockout of MAVS has little effect on virus entry compared with that in control cells (data not shown). To further analyze how U26 affected MAVS expression, we treated U26-expressing cells with either MG132 (a proteasome inhibitor) or chloroquine (a lysosome inhibitor) and found that MAVS expression could be rescued by MG132 treatment but not chloroquine treatment. These results indicated that MAVS reduction occurs mainly through the proteasome pathway rather than the lysosomal pathway (Fig. 4C). These results suggested that MAVS was ubiquitinated for degradation when it was coexpressed with HHV-6B U26. To further investigate U26-dependent ubiquitination of MAVS, we coexpressed MAVS (tagged with Flag), U26, and different types of ubiquitin (wild and K63 and K48 tagged with HA) and then prepared cell lysates for coimmunoprecipitation analysis with anti-Flag antibody. We analyzed the immunoprecipitants with anti-HA antibody and found that MAVS was mainly modified with K63-linked but not K48-linked ubiquitin (Fig. 4D). We also examined whether HHV-6B U26 expression affected endogenous MAVS expression by transfecting HEK293T cells with the U26 expression plasmid alone. We found that U26 inhibited MAVS expression in U26-expressing cells by Western blotting (Fig. 4E). Furthermore, when we used shRNA-expressing lentivirus to knock down U26 during HHV-6B infection, endogenous MAVS expression was significantly upregulated (Fig. 4F).

### U26 inhibits the RLR/MAVS/IRF3 signaling pathway.

**FIG 5:**
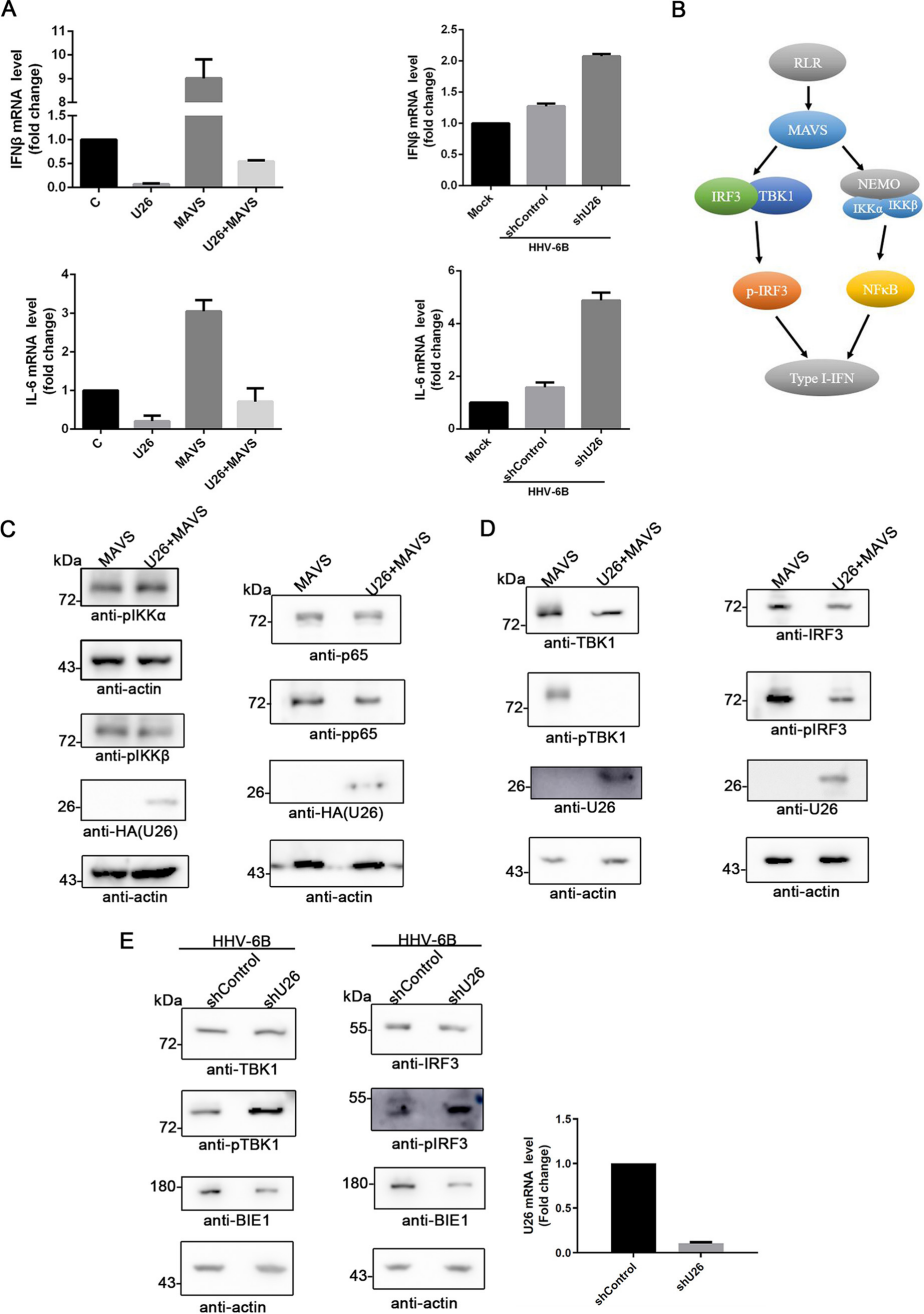
U26 inhibits the RLR/MAVS/IRF3 signal pathway. (A) U26 affected the production of antiviral factors. (Left) HEK293T cells were transfected with plasmids encoding Flag-tagged MAVS and/or U26 and incubated for 24 h. C, control. (Right) Control shRNA- or U26 shRNA-expressing MT4 cells were infected with HHV-6B and harvested at 24 h postinfection. Antiviral factor (IFN-β and IL-6) mRNA expression levels were analyzed by RT-qPCR at the transfection (left) and infection (right) levels. (B) Schematic diagram of the RLR/MAVS signaling pathway. (C) U26 had a minor effect on the NF-κB signaling pathway. HEK293T cells were transfected with Flag-tagged MAVS and HA-tagged U26 and then immunoblotted with the indicated antibodies. (D and E) U26 inhibited MAVS-induced TBK1 and IRF3 phosphorylation. (D) Cell lysates of HEK293T cells expressing Flag-tagged MAVS alone or together with U26 were subjected to immunoblotting with the indicated antibodies. (E) Control shRNA- or U26 shRNA-expressing MT4 cells were infected with HHV-6B. (Left) At 24 h postinfection, IRF3 phosphorylation in these cells was detected by immunoblot analysis. (Right) U26 knockdown efficacy was confirmed by RT-qPCR.

Since U26 downregulated MAVS expression, we hypothesized that mitochondrion-located U26 could negatively regulate the MAVS-mediated signal pathway, thereby inhibiting the production of IFNs and other cytokines. To test this hypothesis, we expressed U26 in HEK293T cells and measured the expression of MAVS-mediated antiviral factors by RT-qPCR. We found that IFN-β and IL-6 cytokine expression was dramatically reduced (Fig. 5A, left). In HHV-6-infected cells, U26 knockdown also resulted in the upregulation of the expression of these two cytokines (Fig. 5A, right). These results indicate the HHV-6B U26 can significantly inhibit the activation of the MAVS-mediated signal pathway. There are two major signal pathways for MAVS to induce cytokine expression. One is the IKK/NF-κB pathway, and the other is the TBK1/IRF3 pathway (Fig. 5B). To further examine which signaling pathway was affected by U26, we analyzed the phosphorylation form of key molecules in these pathways by immunoblot assays. We transfected HEK293T cells with the plasmid for Flag-tagged MAVS expression alone or together with U26 and tested the phosphorylation form of IKKα, IKKβ, and NF-κB in these cells. We found that U26 had minor effects on these proteins’ phosphorylation, which meant that U26 might have a slight effect on this signaling pathway (Fig. 5C). However, when checking the other signaling pathway, we found that U26 dramatically reduced the expression and phosphorylation of TBK1 and IRF3 (Fig. 5D). To confirm U26 inhibition of the TBK1/IRF3 signal pathway, we used U26 shRNA-expressing lentivirus to knock down U26 expression in MT4 cells during HHV-6B infection and then analyzed the phosphorylation of these proteins. The results showed that the phosphorylation of TBK1 and IRF3 was upregulated in U26 knockdown cells compared with that in control cells (Fig. 5E). These data indicated that U26 negatively regulated the RLR/MAVS signal cascade mainly through the TBK1/IRF3 pathway.

### U26 affects mitochondrial membrane potential.

**FIG 6:**
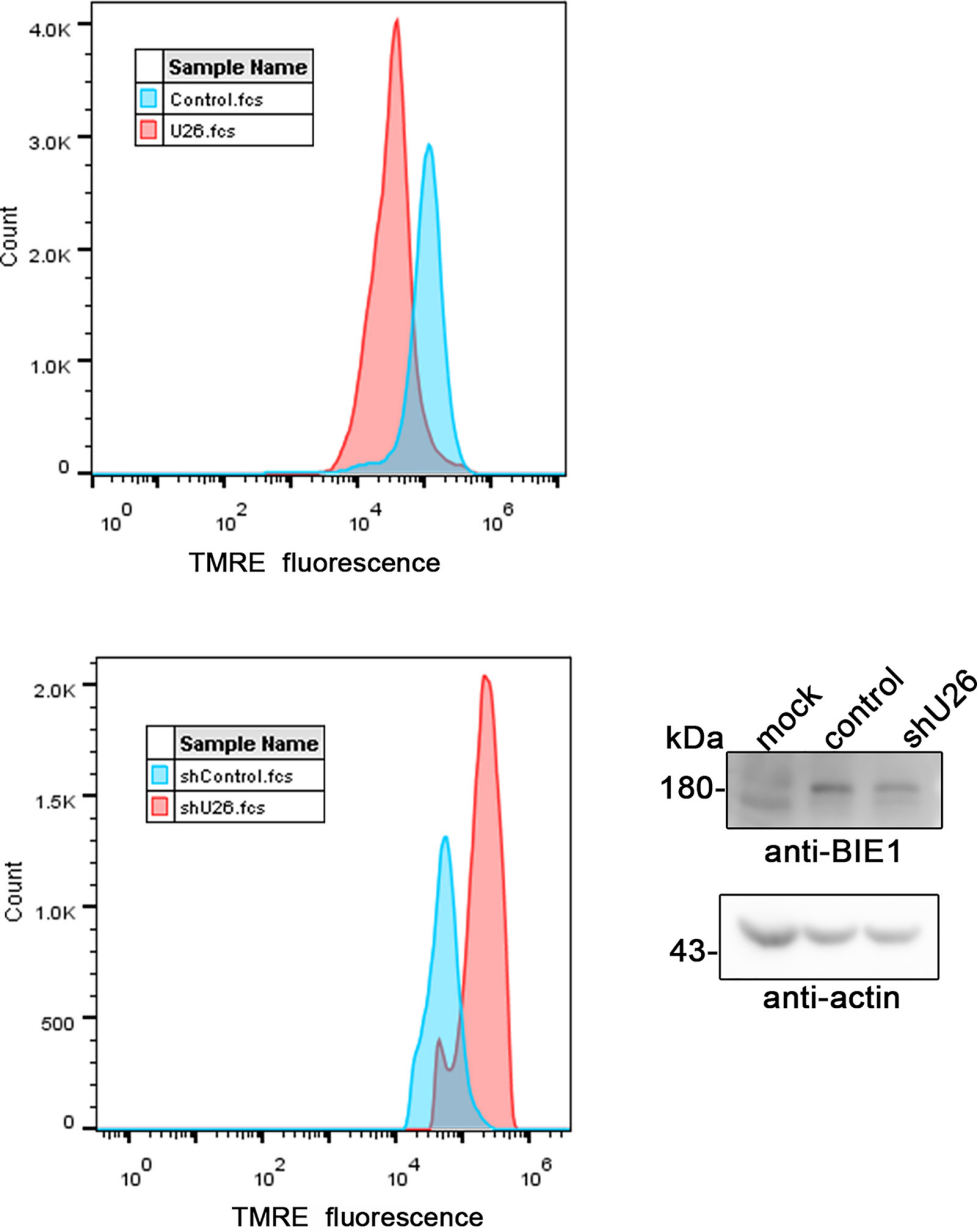
U26 affects mitochondrial membrane potential. (Top) Target cells were transfected with Flag-tagged U26 and incubated for 24 h. (Bottom) Control shRNA- or U26 shRNA-expressing MT4 cells were infected with HHV-6B and harvested at 24 h postinfection. Cells were incubated with TMRE at room temperature for 5 min in the dark, and ΔΨm was then detected using a flow cytometer.

Previous studies have reported that mitochondrial membrane potential (ΔΨm) correlates with MAVS-mediated mitochondrial antiviral responses (36). Viral proteins that aggregate in mitochondria can fragment mitochondria. Mitochondrial fragmentation leads to ΔΨm loss (25, 37). The decrease of ΔΨm further accelerates mitochondrial fragmentation and MAVS translocation (28, 36). We hypothesized that U26 may affect ΔΨm to leak MAVS from mitochondria and then suppress the host innate immune response. To verify this hypothesis, we investigated the mitochondrial membrane potential using a ΔΨm-sensitive dye (tetramethylrhodamine ethyl ester [TMRE]) in U26-expressing cells and U26 knockdown cells infected with HHV-6B. The result showed that ΔΨm was reduced in U26-expressing cells compared with the control cells (Fig. 6, top). In U26 knockdown cells, ΔΨm obviously increased (Fig. 6, bottom). These data indicated that U26 was involved in ΔΨm disruption.

## DISCUSSION

As a mitochondrial membrane protein, MAVS functions as a critical adaptor for the RLR signaling pathway in which it links upstream recognition to downstream signal activation (38, 39). Antiviral functions of RLR for many RNA viruses have been reported (40, 41). Recently, several studies showed that RLR also functions during DNA virus infections (25, 42, 43). Targeting MAVS for viral immune evasion has also been reported during a set of virus infections (28, 44, 45). Here, we report a novel HHV-6B U26-targeted MAVS signaling pathway for inhibition of innate antiviral responses. We elucidated U26 function and analyzed the role of MAVS during HHV-6B infection.

We started our study from HHV-6B U26 functional analyses. The U26 gene is unique to the *Roseolovirus* genus with unknown function in the betaherpesvirus subfamily. Our kinetics analyses revealed that U26 was expressed at an early stage during HHV-6B infection (even at 8 h postinfection) (Fig. 2A), indicating that it should be classified as an immediate early (IE) gene.

Herpesvirus IE genes could function either in nuclei as transcription-associated factors promoting viral gene expression or in the cytosol as disruptors of host antiviral responses during incoming virus propagation (46–49). Our analysis revealed that U26 was expressed in the cytosol and mitochondria (Fig. 3). Several viral proteins expressed in mitochondria could affect the RNA-induced RLR/MAVS signaling pathway (50, 51). We explored whether U26 functioned in a similar way in mitochondria. We found that U26 expression induced MAVS degradation by the proteasome pathway (Fig. 4D and E). Furthermore, we also found that TBK1/IRF3 but not NF-κB activation was affected by U26 expression (Fig. 5).

As to how U26 induced MAVS degradation, we tested the possibility that U26 directly interacted with MAVS. However, the interaction was not confirmed (data not shown), thus offering other possible mechanisms. Several viral proteins jeopardize mitochondrion integrity when they are located in it, which results in the release of MAVS from mitochondria and the degradation of MAVS. For example, the influenza A virus protein PB1-F2 almost completely translocates into the mitochondrial inner membrane space via Tom40 channels, which accelerates mitochondrial fragmentation due to reduced mitochondrial inner membrane potential (37). The hepatitis C virus (HCV) NS3/4A protease cleaves MAVS rapidly, resulting in the release of MAVS from the mitochondrial membrane, destabilization, and loss of its interaction with RIG-I, which finally disables the antiviral signaling pathway (52). The KSHV tegument protein ORF33 interacts with MAVS, which results in the dephosphorylation of MAVS by a host factor, PPM1G, and leads to immunosuppression. The HCMV US9 protein accumulates in mitochondria, which could attenuate the mitochondrial membrane potential and lead to the promotion of MAVS leakage from the mitochondria. Our results showed that one of the mechanisms for U26-induced MAVS degradation is by affecting MAVS function by disrupting mitochondrial integrity (Fig. 6).

Activation of the RLR/MAVS signaling pathway needs the stimulation of pathogen-associated molecular patterns (PAMPs) or damage-associated molecular patterns (DAMPs) (40, 53, 54). However, the possible molecular mechanisms involving RLR/MAVS signaling pathway activation during HHV-6B still need further investigations. For DNA virus infections, several possible mechanisms exist. In some previous studies, it has been reported that RNA polymerase III (Pol III) can mediate double-stranded DNA (dsDNA) recognition by RNA intermediates, which in turn can be recognized by RIG-I and MAVS (42, 43, 55). This is a potential mechanism by which RIG-I could respond to DNA virus infections, via RNA intermediates. During HSV-1 infection, 5S RNA pseudogene transcripts, particularly RNA5SP141, contribute to RIG-1 recognition and activation, possibly because of mislocation of these transcripts in the cytoplasm and downregulation of their binding partners in infected cells. In the case of lytic KSHV infection, 5′-PPP-bearing vault RNAs accumulate because of the reduced level of DUSP11, which could remove two phosphates from the 5′ end of vault RNAs. Moreover, it has been reported that the Epstein-Barr virus (EBV) genome has multiple regions encoding RNA molecules that function as potential virus ligands binding to RIG-I and stimulating RIG-I-dependent but RNA Pol III-independent IFN-β signaling (56). Further research is needed to determine what kind of RNA (cellular or viral) is recognized by RLR/MAVS in HHV-6B infection.

Many RNA and DNA viruses have developed mechanisms to perturb the host IFN response. Based on these findings, we suggest that HHV-6 U26 targets MAVS signaling to evade viral infection in host cells. The virus-host innate immune interaction represents a critical evolutionary battleground that shapes the outcome of viral infection. Therefore, an improved understanding of pU26 may facilitate the development of novel antiviral therapeutics for HHV-6 infection. The molecular mechanism of U26-influenced immune evasion is still unclear and needs to be investigated in future studies.

## MATERIALS AND METHODS

### Cells and virus.

T cell lines (MT4 cells) were cultured in RPMI 1640 medium supplemented with 10% fetal bovine serum (FBS). Human embryonic kidney HEK293T cells and the cervical cancer cell line HeLa were cultured in Dulbecco’s modified Eagle’s medium (DMEM) with 10% FBS. Umbilical cord blood mononuclear cells (CBMCs) were isolated by using Ficoll (Hao Yang Biological Manufacture Co.) and cultured in RPMI 1640 medium containing 10% FBS, phytohemagglutinin (5 μg/ml) (Sigma), and interleukin-2 (2 ng/ml) (Sigma). All culture media contained penicillin (12,000 U/ml), streptomycin (10,000 U/ml), and gentamicin (4,000 U/ml). All cells were grown at 37°C in humidified air with 5% CO_2_. HHV-6B (Z29 strain) was propagated in CBMCs, and cell-free virus was prepared as follows. When HHV-6B-infected CBMCs showed >80% infection (based on the cytopathic effect on infected cells), the cells were lysed by two freeze-thaw cycles. The lysate was centrifuged at 300 × *g* for 5 min. The supernatant was titrated and used as cell-free virus (57).

### 5′ RACE and 3′ RACE.

To identify the ends of U26 transcripts, polyadenylated RNA was isolated from MT4 cells infected with HHV-6B. RACE was performed using a SMARTer RACE cDNA amplification kit (Clontech). For 3′ RACE, U26 was amplified by PCR with the primers 5′-ACCCTCGAGAATTCGCCACCATGCGTCGTCTGACAG-3′ and 5′-CTAATACGACTCACTATAGGGCAAGCAGTGGTATCAACGCAGAGT-3′. For 5′ RACE, U26 was amplified by PCR with the primers 5′-CTAATACGACTCACTATAGGGCAAGCAGTGGTATCAACGCAGAGT-3′ and 5′-ACAGTCGACTCGAGTCACACACCAAAATACATG-3′. The PCR products were purified for sequencing.

### Plasmids.

HA-tagged U26 was amplified by PCR with the primers 5′-ACAGAATTCGCCACCATGCGTCGTCTGACAGATAG-3′ and 5′-ACACTCGAGTTAAGCGTAATCTGGAACATCGTATGGGTACACACCAAAATACATGC-3′ and then cloned into the pCAGGS vector to generate pCAGGSU26HA. To further add an N-terminal Flag tag to U26, it was amplified from pCAGGSU26HA with the primers 5′-ACAGATATCGGACTACAAAGACGATGACGACAAGTTTAGAATGACAGAG-3′ and 5′-AGCCAGAAGTCAGATGCTCAAG-3′. The PCR product was digested with EcoRV and ligated with the pFUSE-hIgG1-Fc2 plasmid, which had also been digested with EcoRV. The resultant ligation product was used as the template for secondary amplification using the primers 5′-ACAGTCGACGAAGGAGGGCCACCATG-3′ and 5′-AGCCAGAAGTCAGATGCTCAAG-3′. The PCR product was digested with SacI and XhoI and then cloned into pCAGGS. The resultant plasmid was named pCAGGSU26FlagHA. The plasmids pcDNA3.1Flag-MAVS, pcDNA3.1HA-MAVS, pcDNA3.1Myc-STING, and pcDNA3.1Flag-TBK1 were kindly provided by Jingfeng Wang (Chinese Academy of Medical Sciences). For knockdown experiments, we used a modified pLKO.1 plasmid (58). Oligonucleotide 1 was generated using the primers 5′-CCGGGCGTTTCTCCTGCCGTTTACTCGAGAGTAAACGGCAGGAGAAACGTTTTTT-3′ and 5′-AATTAAAAAACGTTTCTCCTGCCGTTTACTCTCGAGTAAACGGCAGGAGAAACGC-3′, and oligonucleotide 2 was generated using the primers 5′-CCGGGGATGGATGATACGATCATCTCGAGAGATGATCGTATCATCCATCCTTTTTT-3′ and 5′-AATTAAAAAAGGATGGATGATACGATCATCTCTCGAGATGATCGTATCATCCATCC-3′. Both oligonucleotides 1 and 2 used for U26 knockdown were cloned into the EcoRI and AgeI sites of the modified pLKO.1 vector.

### Antibodies.

A RIG-I pathway antibody sampler kit (catalog number 8348) was purchased from Cell Signaling Technology. The antibody for Tom20 (catalog number AF1717) was purchased from Beyotime. Mouse monoclonal antibodies (MAbs) to Flag (catalog number F1804) and HA (catalog number H3663) tags were purchased from Sigma-Aldrich. To generate the antibodies against HHV-6B IE1and gB proteins, IE1and gB were purified from Escherichia coli transformed with an IE1 or gB expression plasmid as described previously (59, 60). Mice were immunized with the IE1 protein, and the animal sera were used as the polyclonal antibody for IE1. Mice were immunized with gB protein, and MAbs for gB were produced. To generate the antibodies for U26, a plasmid (pGEX-4T-1U26NTD) for the expression of connected nontransmembrane domains in U26 was synthesized by General Biosystems (China), and the recombinant U26 protein was expressed and purified from Escherichia coli, which had been transformed with the plasmid pGEX-4T-1U26NTD. Mice were immunized with the U26 protein, and the animal sera were used as the polyclonal antibody for U26.

### Western blotting.

Samples were lysed with radioimmunoprecipitation assay (RIPA) buffer (Thermo Scientific), separated by SDS-PAGE, electrotransferred onto polyvinylidene difluoride (PVDF) membranes, and reacted with primary antibodies. Reactive bands were detected using a horseradish peroxidase-linked secondary conjugate (Santa Cruz) and visualized using enhanced chemiluminescence (ECL) reagents.

### Coimmunoprecipitation.

Antibodies were bound to protein G-Sepharose (GE Healthcare) by incubation at 4°C for 8 h and then cross-linked to protein G-Sepharose with dimethyl pimelimidate (DMP; Thermo Scientific) according to the manufacturer’s instructions. Cells were lysed in TNE buffer (10mM Tris-HCl [pH 7.8], 0.15 M NaCl, 1 mM EDTA, and 1% NP-40) containing a protease inhibitor cocktail (Sigma-Aldrich) for 30 min at 4°C and subsequently centrifuged at 13,000 × *g* for 1 h at 4°C. The supernatant was added to the appropriate protein G-Sepharose-bound antibody and incubated at 4°C for 8 h. After washing, the bound proteins were eluted with 0.1 M glycine (pH 2.5) and neutralized with 1 M Tris-HCl (pH 8.8) to pH 7.0 to 7.4. The samples were prepared for gel electrophoresis and Western blot detection with the indicated antibodies.

### Ubiquitination assay.

To analyze MAVS ubiquitination, HEK293T cells were transfected with different combinations of plasmids expressing Flag-MAVS, HA-ubiquitin (wild type [WT]), HA-ubiquitin (K48), HA-ubiquitin (K63), and U26. The cell extracts were immunoprecipitated with anti-Flag (MAVS) antibody and analyzed by Western blotting with anti-HA (ubiquitin).

### Immunofluorescence confocal microscopy.

HEK293T cells were fixed with 4% formaldehyde (Beyotime, China) for 20 min at room temperature. Cells were permeabilized and blocked using phosphate-buffered saline (PBS) with 0.3% Triton X-100 and 5% bovine serum albumin (BSA) for 1 h at room temperature. The cells were stained with primary antibodies (indicated in the figures) for 30 min at 37°C, followed by incubation with fluorescent-dye-conjugated secondary antibodies. Nuclei were stained with 4′,6-diamidino-2-phenylindole (DAPI). Samples were mounted in hydrosoluble mounting medium (Boster, Wuhan). Confocal images were acquired using the Zeiss LSM710 confocal laser scanning microscope.

### Generation of knockout MAVS cell lines.

CRISPR-Cas9 technology was used to knock out the MAVS gene in target cells. Guide RNA (gRNA) sequences targeting the MAVS gene were analyzed using the CRISPR gRNA design tool (https://www.atum.bio/eCommerce/cas9/input). The oligonucleotide for MAVS knockout was generated using forward (F) primer 5′-CACCGCTGCCTCACAGCAAGAGACC-3′ and reverse (R) primer 5′-AAACGGTCTCTTGCTGTGAGGCAGC-3′ and subsequently cloned into the lentiCRISPRv2 vector (Addgene). The resultant plasmid was cotransfected with packaging plasmids (pCAG-HIV-gag and pCMV-VSV-G-RSV-Rev) into HEK293T cells. Three days after transfection, the culture supernatant was harvested and concentrated at 2,000 × *g* for 10 min to remove cell debris and then centrifuged at 20,000 × *g* for 90 min at 4°C. Lentiviral particles were collected and used to transduce MT4 cells. The transduced cells were selected in culture medium with puromycin (0.5 μg/ml).

### RT-qPCR and real-time PCR.

Total RNAs were extracted from cells with TRIzol reagent according to the manufacturer’s instructions (Tiangen, China). DNase I (Thermo Scientific, USA) was used to digest single- and double-stranded DNAs. Reverse transcription-quantitative PCR (RT-qPCR) was performed to evaluate the mRNA expression levels of the relevant genes. Typically, 1 μg of total RNA was reverse transcribed into cDNA by using a reverse transcription kit (TaKaRa, Japan).

Real-time PCR was performed to evaluate viral genome quantification. Total DNA was extracted from infected cells and culture medium using a nucleic acid extraction kit (catalog number DP304; Tiangen). RT-qPCR and real-time PCR analyses were performed on a 7500 Fast PCR system (Applied Biosystems) using TB green premix Ex*Taq* (TaKaRa). The primers used for RT-qPCR or real-time PCR are as follows: for IFN-β, F primer 5′-TGGAATGAGACTATTGTTGAGAA-3′ and R primer 5′-ATTTCCACTCTGACTATGGTC-3′; for IL-6, F primer 5′-TTCTCCACAAGCGCCTTCGGTC-3′ and R primer 5′-TCTGTGTGGGGCGGCTACATCT-3′; for U26, F primer 5′-TCTCGGTCTTGCTAAGGGTG-3′ and R primer 5′-CAGATCATGACCACTGCAGA-3′; for U22, F primer 5′-CGCTCGGAAAGGAAACATTA-3′ and R primer 5′-AAGTGGAACTGCTTGGTGGC-3′; and for actin, F primer 5′-TGGCACCCAGCACAATGAA-3′ and R primer 5′-CTAAGTCATAGTCCGCCTAGAAGCA-3′.

### Mitochondrion purification.

A mitochondrial isolation kit (Beyotime, China) was used to isolate cytosolic and mitochondrial fractions from cultured cells. Briefly, 2 × 10^8^ pelleted cells were resuspended with mitochondrial isolation reagent. The cells were broken with ultrasound for 10 s and centrifuged at 800 × *g* for 10 min at 4°C. The supernatant was centrifuged again at 13,000 × *g* for 15 min at 4°C. Next, the supernatant was saved as the cytosol. The pellet containing the mitochondrial fraction was analyzed by Western blotting.

### Flow cytometry.

To detect mitochondrial membrane potential, cells were resuspended at a concentration of 5 × 10^5^ cells/ml in culture medium containing 150 nM TMRE (MedChemExpress, USA) and incubated for 5 min at room temperature in the dark. To demonstrate the fluorescence specificity of TMRE, carbonylcyanide 4-(trifluoromethoxy)phenylhydrazone (FCCP; 5 mM final concentration) was added to an aliquot of untreated cells, and the mixture was incubated for 5 min at room temperature in the dark. The cells were immediately analyzed by flow cytometry (CytoFLEX; Beckman).
